# Afatinib for Advanced Non-small Cell Lung Cancer in a Case With an Uncommon Epidermal Growth Factor Receptor Mutation (G719A) Identified in the Cerebrospinal Fluid

**DOI:** 10.3389/fonc.2019.00628

**Published:** 2019-07-23

**Authors:** Chunhua Ma, Chuoji Huang, Dongjiang Tang, Xin Ye, Zhi Li, Renzhong Liu, Ning Mu, Jing Li, Rong Jiang, Juncheng Zhang

**Affiliations:** ^1^Department of Intervention, Tianjin Huanhu Hospital, Tianjin Key Laboratory of Cerebral Vascular and Neurodegenerative Disease, Tianjin, China; ^2^Zhuhai SanMed Biotech Ltd., Zhuhai, China; ^3^Joint Research Center of Liquid Biopsy in Guangdong, Hong Kong and Macao, Zhuhai, China; ^4^Zhuhai Livzon Gene Diagnostics Ltd., Zhuhai, China

**Keywords:** cerebrospinal fluid, G719A mutation, leptomeningeal metastases, NSCLC, afatinib

## Abstract

Few previous studies of patients with non-small cell lung cancer (NSCLC) and leptomeningeal metastases have used liquid biopsy of cerebrospinal fluid (CSF) to identify epidermal growth factor receptor (EGFR) mutations and guide therapy. A 34-year-old male patient with NSCLC and leptomeningeal metastases was admitted to the Interventional Radiology Department, Tianjin Huanhu Hospital on 18th April 2018 after showing no response to chemoradiotherapy. On admission, the patient was in critical condition with an estimated survival <1 month. A ventriculoperitoneal shunt was placed in the right lateral ventricle. The CSF level of carcinoembryonic antigen (CEA) was 9,869 ng/mL. Next-generation sequencing (NGS) of the CSF revealed an EGFR G719A mutation (frequency: 55.63%), whereas sequencing of circulating tumor DNA or cells in the peripheral blood identified no clinically significant mutations. Afatinib therapy was initiated based on the NGS results. During follow-up, the patient's symptoms improved, ventricular dilatation lessened, and pulmonary lesions decreased in size. At the last follow-up (7 months), the patient continued to show a good response to afatinib therapy with minimal adverse effects. This is the first clinical study to report the use of simultaneous genetic testing of CSF and peripheral blood to guide the successful implementation of afatinib therapy in a patient with NSCLC and leptomeningeal metastases. Notably, NGS of CSF was superior to genetic testing of peripheral blood at identifying an uncommon EGFR mutation (G719A) in a patient with NSCLC and leptomeningeal metastases.

## Introduction

Around 3–5% of patients with advanced stage (IIIb–IV) lung adenocarcinoma develop leptomeningeal metastases (1). New technologies, e.g., next-generation sequencing (NGS), have improved our understanding of non-small cell lung cancer (NSCLC) pathogenesis, and drug resistance (2). Epidermal growth factor receptor (EGFR) mutation detection traditionally relies on fixed tissue samples and polymerase chain reaction (PCR) (3). However, the Food and Drug Administration (FDA) recently approved liquid biopsy for detecting circulating free tumor DNA (cfDNA) in peripheral blood samples (3). Liquid biopsy of cerebrospinal fluid (CSF) using NGS can identify unique genetic profiles not detected in plasma (3). Nevertheless, no studies have utilized CSF liquid biopsy to detect gene mutations and guide therapy in patients with NSCLC and leptomeningeal metastases.

Since the discovery of EGFR mutations that promote tumor proliferation/metastasis, the development of tyrosine kinase inhibitors (TKIs) as targeted therapeutics has radically changed NSCLC treatment (2, 4). First-generation TKIs improve outcomes in patients with EGFR-mutant NSCLC but are less effective in patients with wild-type EGFR, and drug resistance remains an issue (2, 4). Afatinib (BIBW2992) is a second-generation TKI that inhibits EGFR, HER2, and HER4, and ErbB3 phosphorylation (5). Afatinib was approved by the U.S. FDA in 2018 for metastatic NSCLC with uncommon non-drug-resistant EGFR mutations (L861Q, G719X, and/or S768I) (5). However, few studies have reported afatinib use in patients with NSCLC and leptomeningeal metastases, particularly those with uncommon EGFR mutations.

We report the use of CSF liquid biopsy to identify an uncommon EGFR mutation (G719A) in a patient with NSCLC and leptomeningeal metastases. The patient was treated with afatinib, with a good response.

## Case Report

This study was approved by the ethics committee of Tianjin Huanhu Hospital, Tianjin, China. The patient provided informed written consent. The written informed consent was obtained from the participant for the publication of this case report and any potentially identifying images/information. A 34-year-old man with NSCLC and leptomeningeal metastases, showing no obvious improvement after radiotherapy and chemotherapy, was admitted to the Department of Interventional Radiology, Tianjin Huanhu Hospital on 18th April 2018. The initial diagnosis of lung adenocarcinoma was made in May 2016 by chest computed tomography (CT) and histopathology. Leptomeningeal metastasis was diagnosed in June 2017 based on linear high-intensity signals in the medulla oblongata, pons, and ventral and dorsal midbrain on cranial enhanced magnetic resonance imaging (MRI). In May 2016, NGS had shown very low variant allele frequencies for G719A in exon 18 (0.12%) and C797S in exon 20 (0.04%) of the EGFR, but interpreted as false positives. In September 2017, no EGFR-sensitive mutations were detected in blood assayed by droplet digital PCR (dd-PCR). Prior to admission to our hospital, the patient had received 6 cycles of docetaxel/cisplatin, 1 cycle of gemcitabine/carboplatin, 10 cycles of pemetrexed, 4 cycles of temozolomide, whole-brain radiotherapy, bevacizumab, and semustine. The patient denied a history of hypertension, diabetes mellitus, or coronary heart disease; a family history of cancer; or addiction to alcohol or tobacco.

**Figure 1:**
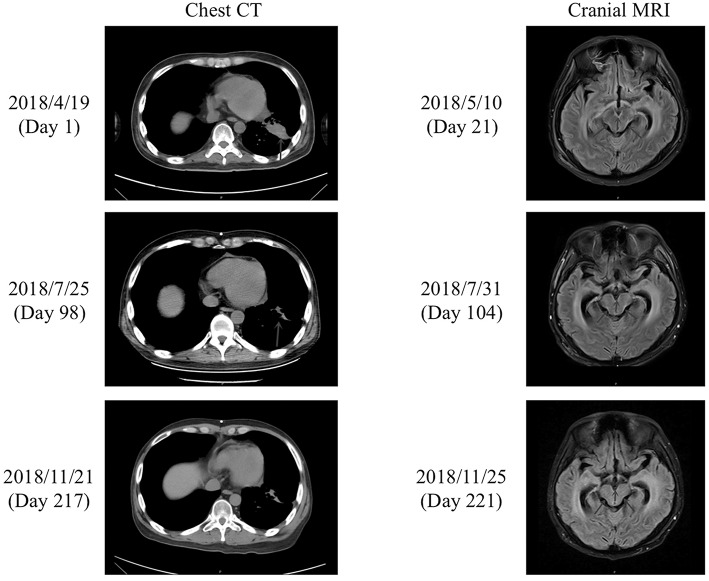
Chest CT and cranial MRI. **(Left)** Chest CT performed on the day of admission (day 1) and after treatment with afatinib for 71 days (day 98) and 190 days (day 217). The lung lesions (blue arrows) were reduced in size after treatment with afatinib for 71 days (day 98), and the effect was maintained after a further 119 days of treatment (day 217). **(Right)** Enhanced cranial MRI T2 FLAIR scans performed on day 21 and after treatment with afatinib for 77 days (day 104) and 194 days (day 221). The cranial MRI scan on day 21 showed high-intensity linear shadows in the medulla oblongata, pons, and ventral and dorsal midbrain, which were considered to be leptomeningeal metastases. The cranial scan on day 104 revealed similar abnormalities to those detected on day 21. However, the abnormal signals in the medulla oblongata, pons, and ventral and dorsal midbrain on day 221 were decreased in comparison to those on days 21 and 104.

On admission, the patient was in a critical condition [Karnofsky Performance Score (KPS), 10–20 points], and prognosis was <1 month. Routine blood tests revealed the following (normal range in brackets): white blood cell count, 5.80 × 10^9^/L (3.5–9.5 × 10^9^/L); red blood cell count, 3.20 × 10^12^/L (4.3–5.8 × 10^12^/L); hemoglobin, 103 g/L (130–175 g/L); platelet count, 135 × 10^9^/L (125–350 × 10^9^/L); albumin, 34.7 g/L (40–55 g/L); urea, 3.3 mmol/L (3.1–8 mmol/L); creatinine, 39.6 μmol/L (57–97 μmol/L); glucose, 4.3 mmol/L (3.89–6.12 mmol/L); potassium, 3.5 mmol/L (3.5–5.3 mmol/L); sodium, 143 mmol/L (137–147 mmol/L); chloride, 102 mmol/L (96–108 mmol/L); and alanine transaminase, 31 U/L (9–50 U/L). Chest CT on day 1 revealed lung lesions (Figure 1), while cranial CT showed dilation of the supratentorial ventricular system and widening of the cerebral sulcus and cistern. Enhanced cranial MRI T2 fluid-attenuated inversion recovery (FLAIR) scans on day 21 showed high-intensity linear shadows in the medulla oblongata, pons, and ventral and dorsal midbrain, indicating leptomeningeal metastasis (Figure 1). CSF pressure was 20 mmHg on lumbar puncture.

**Figure 2:**
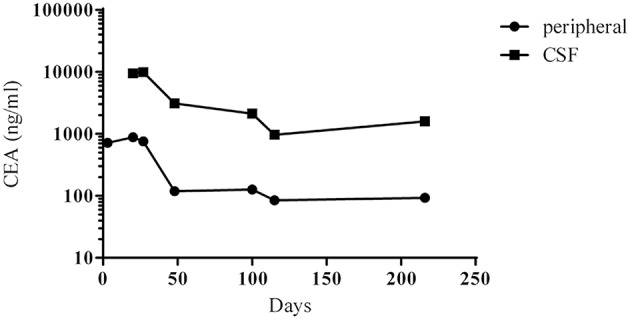
Changes in the concentrations of CEA in peripheral blood and CSF between day 1 (the initial admission) and day 216. Therapy with afatinib was commenced on day 27.

On 21st April 2018 (day 3), a ventriculoperitoneal shunt was placed in the right lateral ventricle, and carcinoembryonic antigen (CEA) levels in peripheral blood were 717 ng/mL (Figure 2). On 8th May 2018 (day 20), lumbar puncture revealed a CSF pressure of 8 mmHg, and CSF CEA levels were 9,470 ng/mL (Figure 2). Ten microliters of CSF drainage and 10 mL of peripheral blood were sent for genetic testing (NGS), and methotrexate (8 mg) and dexamethasone (1 mL) diluted in normal saline (8 mL) were injected through the lumbar puncture needle. On 15th May 2018 (day 27), NGS results for the CSF revealed an EGFR G719A mutation at 55.6% frequency (Table 1), with EGFR mutations undetectable in peripheral blood; CSF CEA levels were 9,869 ng/mL. Based on NGS results, afatinib therapy [30 mg per os (po) qd] was initiated. On 5th June 2018 (day 48), peripheral blood CEA had fallen to 118.5 ng/mL, and lumbar puncture showed a CSF pressure of 10 mmHg. Intrathecal chemotherapy was performed using methotrexate (8 mg) and dexamethasone (1 mL) in normal saline (8 mL). On 12th June 2018 (day 55), the patient's condition had improved sufficiently (KPS, 60 points) to allow discharge.

**Table 1:** Genetic mutations detected by liquid biopsy of CSF and peripheral blood.

**Date**	**Gene**	**Amino acid site**	**Allele frequency**	**Sample type**
2018/5/8	EGFR	p.(G719A)	55.63%	CSF
2018/8/10	EGFR	p.(G719A)	22.21%	CSF
	NRAS	p.(G12V)	0.62%	CSF
	EGFR	p.(G873R)	0.65%	Peripheral blood cfDNA
2018/11/20	EGFR	p.(G719A)	23.10%	CSF
	EGFR	p.(G873R)	2.15%	Peripheral blood cfDNA

*cfDNA, circulating free DNA*.

After discharge, the patient continued to take afatinib (30 mg po qd). Headache and dizziness improved substantially, suggesting effective treatment. Follow-up on 25th July 2018 (day 98) demonstrated reduced pulmonary lesions (chest CT; Figure 1) and improved supratentorial ventricular dilation. On 27th July 2018 (day 100), lumbar puncture showed a CSF pressure of 15 mmHg and CSF CEA levels of 2,111 ng/mL. Intrathecal chemotherapy was performed using methotrexate (8 mg) and dexamethasone (1 mL) in saline (9 mL). CSF and peripheral blood samples were collected for repeat genetic testing; the pressure of the ventriculoperitoneal shunt device was adjusted to 1.0, with afatinib dose increased to 40 mg qd. The main adverse effects during afatinib therapy were face and trunk rashes, requiring no treatment. On 10th August 2018 (day 114), NGS results for the CSF revealed an EGFR G719A mutation with a frequency of 22.2% (around 60% less than pre-afatinib therapy) and a very low frequency (0.6%) of NRAS G12V mutation, which is associated with drug resistance (Table 1). EGFR G719A mutation was undetectable in peripheral blood, although EGFR G873R mutation was identified at low frequency (Table 1). On 20th November 2018 (day 216), peripheral blood CEA level was 93 ng/mL; CSF CEA level was 1,590 ng/mL (Figure 2), with a CSF pressure of 6 mmHg, indicating maintenance of the response to afatinib therapy. NGS results for the CSF revealed an EGFR G719A mutation frequency of 23.1%, comparable to that measured on 10th August 2018, while liquid biopsy of peripheral blood indicated increased frequency of EGFR G873R mutation to 2.2% (Table 1). The patient reported intermittent headache likely from meningeal metastasis. However, the patient had good clinical condition (KPS score, 70–80 points). At last follow-up (1st December 2018; day 227), the patient remained in good condition. Although the patient reported mild rash, diarrhea, and oral mucositis, no serious adverse reactions to afatinib had occurred. Thus, the patient had progression-free survival (PFS) > 7 months.

## Discussion

This case report describes the successful use of afatinib in a patient with NSCLC and leptomeningeal metastases who had an uncommon mutation of EGFR (G719A) detected by NGS of the CSF. A novelty in this study was that simultaneous genetic testing of CSF and peripheral blood was performed, and the EGFR mutation was identified in CSF but not in blood samples. Another notable aspect was that the patient responded well to afatinib, suggesting that this therapy might be effective in patients with NSCLC and leptomeningeal metastases harboring EGFR G719A mutation. Additionally, this study highlights the importance of genetic testing to detect mutations in unusual loci. Previously, genetic testing of the CSF (mainly PCR based) has only been used to identify common drug-sensitive loci, but detecting unusual loci may offer new treatment opportunities. Therefore, in patients with NSCLC and leptomeningeal metastases, liquid biopsy of CSF may be better at detecting EGFR mutations relevant to metastatic lesions than peripheral blood. Future studies should extend our observation and identify uncommon mutations associated with a good response to treatment.

We utilized a patent-protected cell preservation liquid (patent no. CN201710442744.2, Zhuhai Livzon Cynvenio Diagnostics Ltd.) to transport peripheral blood and CSF to ensure high-quality samples for sequencing. Perhaps the most novel aspect of this study was NGS application to CSF samples to identify a mutation not detected in peripheral blood. Furthermore, to our knowledge, this is the first study using NGS of CSF to identify a rare EGFR mutation in a patient with brain metastases. Liquid biopsy of peripheral blood has been proposed as a novel approach to facilitate precision therapy in NSCLC (6). However, plasma cfDNA does not necessarily represent tumor DNA from intracranial lesions. Little is known about the mechanisms underlying the development of brain metastases and their acquisition of drug resistance, and liquid biopsy of CSF provides a new method for identifying the contribution of mutations to these mechanisms. Nevertheless, very few studies have used liquid biopsy of CSF to detect EGFR mutations in NSCLC with leptomeningeal metastases. In patients with leptomeningeal metastases from EGFR-mutant NSCLC, liquid biopsy of CSF using NGS can detect distinct genetic profiles to those identified in plasma samples, suggesting that CSF may have an important value in the liquid biopsy of leptomeningeal metastases (7). Consistently, tumor DNA in the CSF better represented genomic changes in brain tumors than that in plasma (8). Furthermore, differences in EGFR mutations between cranial and extracranial samples have also been highlighted (9). Thus, NGS of CSF may provide better insights into the genetic features of brain metastases than peripheral blood. Interestingly, NGS of CSF in this patient also revealed a very low frequency of NRAS G12V mutation, which may be associated with drug resistance. We consider that NGS of CSF has several advantages over peripheral blood in patients with NSCLC and leptomeningeal metastases. First, the blood–brain barrier (BBB) permits only small amounts of tumor DNA and cells to reach the plasma, limiting the value of plasma cfDNA or circulating tumor cells in evaluating intracranial lesions. CSF would better reflect the genetic spectrum of brain tumor lesions. Second, tumor-derived cfDNA in plasma is susceptible to background interference, which is less of an issue for tumor-derived DNA in the CSF due to relatively higher tumor DNA amounts. Third, liquid biopsy of CSF could potentially be used to dynamically monitor the burden of leptomeningeal metastases or novel mutations. Fourth, PCR only detects mutations at default sites; hence, high-throughput NGS may be more appropriate for patients with metastatic NSCLC and a history of targeted therapy who have unknown mutations. Nonetheless, to fully characterize the genetic features of NSCLC and leptomeningeal metastases, it is important to assess CSF and peripheral blood samples simultaneously. This was highlighted in this study by the detection of another uncommon EGFR mutation (G873R) in peripheral blood that showed an apparent increase in frequency between days 114 and 216. Future research characterizing gene mutations by liquid biopsy of CSF and peripheral blood could facilitate the identification of driver and resistance mutations (10) and guide precision therapy for leptomeningeal metastases from NSCLC.

TKIs are more effective for NSCLC with activating EGFR mutations than for NSCLC with wild-type EGFR (11). Furthermore, patients with EGFR mutations may be more prone to brain metastases than those with wild-type EGFR (12). Patients with NSCLC and EGFR mutation are more likely to develop leptomeningeal metastases, and those treated with a TKI had longer overall survival (OS) than those not given a TKI (7). Although first-generation TKIs are effective in patients with NSCLC brain metastases (13), secondary resistance could occur after 9–13 months (14). Secondary resistance can often be overcome by second-generation TKIs, e.g., afatinib (15). However, data are limited regarding the associations of uncommon EGFR mutations with response to TKI therapy. Among patients with NSCLC administered afatinib in the LUX-Lung 2 (phase II), LUX-Lung 3 (phase III), and LUX-Lung 6 (phase III) clinical trials, the objective response rate was 100% in cases with S7681 mutation, 77.8% in patients with G719X mutation, and 56.3% in patients with S7681 mutation (16, 17). Based on these data, the FDA approved afatinib for metastatic NSCLC with unusual EGFR mutations (L861Q, G719X, and/or S768I). These data were supported by a multicenter phase II clinical trial of patients with metastatic or recurrent NSCLC and unusual EGFR mutations who were treated with osimertinib (18). The most common mutations were G719A/C/D/S/X (53% of patients), L861Q (25%), and S768I (22%), and the partial remission rate was 77.8% for patients with L861Q mutation, 52.6% for patients with G719A/C/D/S/X mutation, and 37.5% for patients with S768I mutation (19). The decision to treat our patient with afatinib after the detection of the EGFR mutation was based on the results of these previous studies.

To our knowledge, this is the first report to describe the successful use of afatinib in a patient with leptomeningeal metastases from NSCLC with an uncommon EGFR mutation (G719A) detected in the CSF by NGS. Afatinib was effective in relieving CNS symptoms, reducing the sizes of the tumor lesions and lowering the levels of CEA in peripheral blood and CSF. Importantly, the patient in our study demonstrated a prolonged response to treatment that was well maintained at the last follow-up over 7 months after the initiation of therapy. This is notably longer than a previous study of patients with NSCLC, leptomeningeal metastases, and non-classical EGFR mutations: after treatment with afatinib, median PFS and OS were only 2.0 and 3.8 months, respectively (19). Another study reported that adverse events were less common for an afatinib starting dose of 40 mg/day than for a dose of 50 mg/day (17). We used an even lower starting dose of afatinib (30 mg/day) due to the poor clinical condition of the patient (KPS of 10–20 points) and the potential impact of adverse reactions caused by afatinib. The afatinib dose was increased to 40 mg/day (the recommended dosage) after the patient's clinical condition had improved and the KPS had increased to 60 points. Our patient tolerated afatinib well throughout the course of therapy: the main adverse effects were rashes on the face and trunk, and these required no specific treatment. Importantly, no serious adverse reactions (grade 3 or above) occurred during afatinib therapy, even after the increase in dosage. Although EGFR mutations in exon 18 comprise only a small proportion of all EGFR mutations, the very large number of patients with lung cancer in China means that many would harbor this mutation type. Since there is evidence that NSCLC with mutations in exon 18 of the EGFR is more susceptible to second-generation TKIs than to first- or third-generation TKIs (20), we suggest that afatinib may be a good option for patients with mutations in exon 18 of the EGFR.

## Conclusion

Liquid biopsy of the CSF using NGS may provide more reliable information than liquid biopsy of peripheral blood regarding uncommon EGFR mutations in patients with NSCLC and leptomeningeal metastases, and this information can potentially be used to guide therapy with TKIs. Furthermore, treatment with afatinib may be a good option in patients with NSCLC and leptomeningeal metastases who have uncommon mutations in exon 18 of the EGFR such as G719A.

## Data Availability

All datasets generated for this study are included in the manuscript and/or the supplementary files.

## Ethics Statement

This study was approved by the ethics committee of Tianjin Huanhu Hospital, Tianjin, China. The patient provided informed written consent.

## Author Contributions

CM and CH carried out the studies, participated in collecting data, and drafted the manuscript. CH, JZ, and RJ performed the statistical analysis and participated in its design. DT, XY, JL, NM, ZL, and RL helped draft the manuscript. All authors read and approved the final manuscript.

### Conflict of Interest Statement

The authors declare that the research was conducted in the absence of any commercial or financial relationships that could be construed as a potential conflict of interest.
