# Corrigendum: Afatinib for Advanced Non-small Cell Lung Cancer in a Case With an Uncommon Epidermal Growth Factor Receptor Mutation (G719A) Identified in the Cerebrospinal Fluid

**DOI:** 10.3389/fonc.2019.00938

**Published:** 2019-09-25

**Authors:** Chunhua Ma, Chuoji Huang, Dongjiang Tang, Xin Ye, Zhi Li, Renzhong Liu, Ning Mu, Jing Li, Rong Jiang, Juncheng Zhang

**Affiliations:** ^1^Department of Intervention, Tianjin Huanhu Hospital, Tianjin Key Laboratory of Cerebral Vascular and Neurodegenerative Disease, Tianjin, China; ^2^Zhuhai SanMed Biotech Ltd., Zhuhai, China; ^3^Joint Research Center of Liquid Biopsy in Guangdong, Hong Kong and Macao, Zhuhai, China; ^4^Zhuhai Livzon Gene Diagnostics Ltd., Zhuhai, China

**Keywords:** cerebrospinal fluid, G719A mutation, leptomeningeal metastases, NSCLC, afatinib

In the original article, there was an error. “G719A” was erroneously written as “G791A.”

A correction has been made the ***Case Report*** section, paragraph four:

“After discharge, the patient continued to take afatinib (30 mg po qd). Headache and dizziness improved substantially, suggesting effective treatment. Follow-up on 25th July 2018 (day 98) demonstrated reduced pulmonary lesions (chest CT; Figure 1) and improved supratentorial ventricular dilation. On 27th July 2018 (day 100), lumbar puncture showed a CSF pressure of 15 mmHg and CSF CEA levels of 2,111 ng/mL. Intrathecal chemotherapy was performed using methotrexate (8 mg) and dexamethasone (1 mL) in saline (9 mL). CSF and peripheral blood samples were collected for repeat genetic testing; the pressure of the ventriculoperitoneal shunt device was adjusted to 1.0, with afatinib dose increased to 40 mg qd. The main adverse effects during afatinib therapy were face and trunk rashes, requiring no treatment. On 10th August 2018 (day 114), NGS results for the CSF revealed an EGFR G719A mutation with a frequency of 22.2% (around 60% less than pre-afatinib therapy) and a very low frequency (0.6%) of NRAS G12V mutation, which is associated with drug resistance (Table 1). EGFR G719A mutation was undetectable in peripheral blood, although EGFR G873R mutation was identified at low frequency (Table 1). On 20th November 2018 (day 216), peripheral blood CEA level was 93 ng/mL; CSF CEA level was 1,590 ng/mL (Figure 2), with a CSF pressure of 6 mmHg, indicating maintenance of the response to afatinib therapy. NGS results for the CSF revealed an EGFR G719A mutation frequency of 23.1%, comparable to that measured on 10th August 2018, while liquid biopsy of peripheral blood indicated increased frequency of EGFR G873R mutation to 2.2% (Table 1). The patient reported intermittent headache likely from meningeal metastasis. However, the patient had good clinical condition (KPS score, 70–80 points). At last follow-up (1st December 2018; day 227), the patient remained in good condition. Although the patient reported mild rash, diarrhea, and oral mucositis, no serious adverse reactions to afatinib had occurred. Thus, the patient had progression-free survival (PFS) > 7 months.”

The authors apologize for this error and state that this does not change the scientific conclusions of the article in any way. The original article has been updated.

